# PcG Complexes Set the Stage for Epigenetic Inheritance of Gene Silencing in Early S Phase before Replication

**DOI:** 10.1371/journal.pgen.1002370

**Published:** 2011-11-03

**Authors:** Chiara Lanzuolo, Federica Lo Sardo, Adamo Diamantini, Valerio Orlando

**Affiliations:** 1 Epigenetics and Genome Reprogramming, Dulbecco Telethon Institute, IRCCS Santa Lucia Foundation, Rome, Italy; 2 CNR Institute of Neurobiology and Molecular Medicine, IRCCS Santa Lucia Foundation, Rome, Italy; 3 Neuroimmunology Unit, Santa Lucia Foundation at the Centro Europeo per la Ricerca sul Cervello, Rome, Italy; Max-Planck-Institute of Immunobiology, Germany

## Abstract

Polycomb group (PcG) proteins are part of a conserved cell memory system that conveys epigenetic inheritance of silenced transcriptional states through cell division. Despite the considerable amount of information about PcG mechanisms controlling gene silencing, how PcG proteins maintain repressive chromatin during epigenome duplication is still unclear. Here we identified a specific time window, the early S phase, in which PcG proteins are recruited at BX-C PRE target sites in concomitance with H3K27me3 repressive mark deposition. Notably, these events precede and are uncoupled from PRE replication timing, which occurs in late S phase when most epigenetic signatures are reduced. These findings shed light on one of the key mechanisms for PcG–mediated epigenetic inheritance during S phase, suggesting a conserved model in which the PcG–dependent H3K27me3 mark is inherited by dilution and not by *de novo* methylation occurring at the time of replication.

## Author Summary

During embryonic development, pluripotent cells divide and use their potential to differentiate into a variety of cells with identical genomes but different phenotypes. The emerging concept suggests that the DNA sequence information is not the sole determinant of cell identity. Indeed, epigenetic mechanisms, acting via chromatin organization, control transcriptome complexity and contribute to maintain cell fate. Polycomb-group proteins (PcG) are epigenetic transcriptional regulators that maintaining gene silencing programs through cell division. During S phase, in addition to DNA, the entire epigenome needs to be duplicated. A key question that remains to be addressed is how epigenetic marks are transmitted to subsequent generations. In this study we propose a model for PcG epigenetic inheritance during replication. We found that, during S phase, PcG engagement and characteristic H3K27me3 histone mark deposition on target sites are restricted to a brief interval occurring before DNA replication of the same regions. By increasing the dose of PcG binding the system would prevent potential weakening of silencing control, which is challenged at the time of replication, allowing proper transmission of epigenetic marks to the next generation and preservation of cell identity.

## Introduction

The genes of the Polycomb group (PcG) prevent changes in cell lineage identity by maintaining silenced transcription patterns throughout cell division via chromatin structure [1]. To date, four PcG-encoded protein complexes have been isolated from different organisms: Pho Repressive Complex (PhoRC), Polycomb Repressive Deubiquitinase Complex (PR-DUB), Polycomb Repressive Complex 1 (PRC1) and 2 (PRC2). Biochemical studies revealed that the zinc finger protein Pleiohometic (PHO) of PhoRC is required for PRC2 targeting [2]–[4] while Enhancer of zeste (E(Z)), the Histone Methyl Transferase (HMTase) subunit of PRC2, marks lysine 27 of histone H3 [5]–[8]. This chromatin mark is specifically recognized by PRC1 complex through the chromo-domain present in the Polycomb protein (PC) [5]. PRC1 complex has several catalytic functions believed to be important for transcriptional repression. By electron microscopy, it has been shown that PRC1 induces compaction of defined nucleosomal arrays *in vitro* [9]. Components of PRC1 can also function as E3 ligase for H2A ubiquitylation [10]. On other hand, PR-DUB complex is able to deubiquinate H2A [11] and, interestingly, both activities are required for proper gene silencing *in vivo*. In *Drosophila*, PcG function is mediated by specialized epigenetic DNA modules called Polycomb Response Elements (PREs), which organize repressed PcG target genes at a distance via chromatin structure and nuclear architecture [12]–[16]. Notably, similar *cis*-elements were recently reported in mammals [17], [18]. The characteristic feature of the PcG memory system is the mitotic inheritability of gene expression patterns. However, the mechanism by which PcG proteins maintain repressive chromatin during cell division is poorly understood. In mammals, it has been proposed that PRC2 binds to its own methylation mark H3K27me3 to re-establish epigenetic signatures after replication [19], [20]. In *Drosophila*, by *in vitro* and partially *in vivo* assay, it has been observed that PSC, a chromatin compacting subunit of PRC1 complex, remains bound to chromatin during replication [21]. Such an association suggests that, in principle, epigenetic players could be transferred from maternal to daughter strands. To date however, direct evidence for existence of these models *in vivo* is still lacking. In particular, the time at which the parental marks are imposed and how tightly the process of PcG epigenetic inheritance is coupled to replication have not been determined. To address these questions we used the *D. melanogaster* embryonic Schneider 2 cell line (S2) to analyse replication timing, PcG proteins binding, , H3K27me3 mark deposition, dynamics of PRE mediated higher order structures and transcriptional repression during S phase. Our data suggest a putative conserved mechanism for epigenetic inheritance, identifying a critical time window before replication, during which the PcG memory system sets the stage for subsequent epigenome duplication.

## Results

### Repressed PREs replicate during late S phase

**Figure 1:**
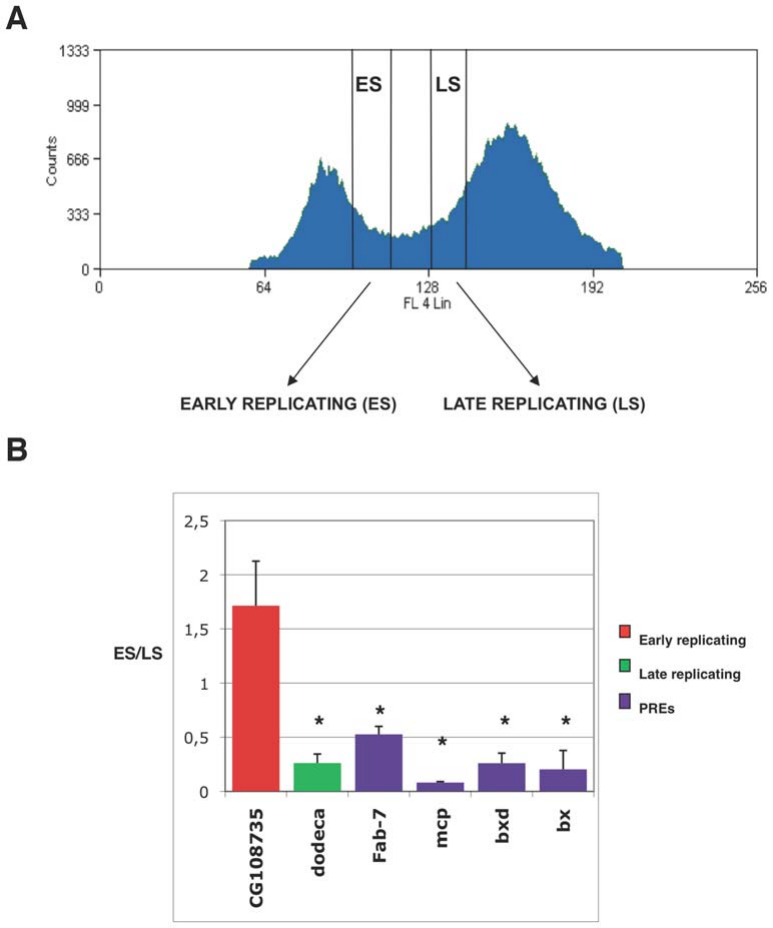
Repressed PREs replicate during late S phase. (A) Experimental strategy to collect cells of the early and late S phase using FACS sorting. Cell-cycle profile of *D. melanogaster* S2 cells after propidium iodide staining. Cells between the G1 and G2 peaks are in S phase. Gates indicate early and late S phase fractions. (B) Replication timing of PREs as measured by quantitative Real Time PCR (qRTPCR). Ratios between the amplified products in early and late S phase are shown. We amplified positive controls for the early and late S phase and gene names correspond to their entries in FlyBase. All data points were generated from an average of five independent experiments. Standard error of the mean is indicated. Two-tailed *t*-test was applied for statistical analysis. Asterisks indicate statistically relevant differences; α = 0.05. *P values*: CG108735/dodeca: *P* = 0.0047; CG108735/*Fab-7: P* = 0.013; CG108735/*mcp: P* = 0.002; CG108735/*bxd: P* = 0.0048; CG108735/*bx: P* = 0.0053.

We first measured BX-C PRE replication timing in S2 embryonic cell line where the homeotic genes of Bithorax Complex (BX-C) are silenced. The relative abundance of nascent DNA synthesised during different fractions of the S phase was determined by bromodeoxyuridine triphosphate (BrdU) labelling and FACS (fluorescence-activated cell sorting) sorting [22]. DNA was prepared from an equal number of cells representing the first and last stages of the S phase, hereafter referred to as “early” and “late” (Figure 1A). BrdU-labelled DNA was immunoprecipitated from these S-phase specific fractions to enrich for genomic sequences that replicate during the labelling period. We then performed quantitative real-time PCR (qRT-PCR), using primers specific for *Fab-7*, *Mcp*, *bxd*, *bx* PREs, and control regions, the latters consisting of *CG108735* gene locus and *dodeca* repeats, which are early and late replicating sequences, respectively [22]. Ratios between the amounts of amplified products in early and late S phase showed that repressed PREs replicate late during S phase (Figure 1B), in agreement with the *Drosophila* genome wide replication timing database [23]. We then repeated the experiment using synchronized S2 cells to confirm that BX-C PRE replication timing was comparable to the FACS-sorted cells. Upon release from hydroxyurea (HU) block (Figure S1A), cells synchronously proceeded through S phase over the next 2 hours (end of S phase). Cells representative of early S phase were pulse-labelled with BrdU at the start of S phase and collected after 1 h from the HU block release, while cells representing late S phase were pulse-labelled after 1 h and collected after 2 h from HU block release. Quantification of the relative amount of PRE sequences after BrdU immunoprecipitation (Figure S1B) confirmed that repressed PREs are late replicating in synchronized S2 cells.

### Higher order interactions are dynamic during replication

**Figure 2:**
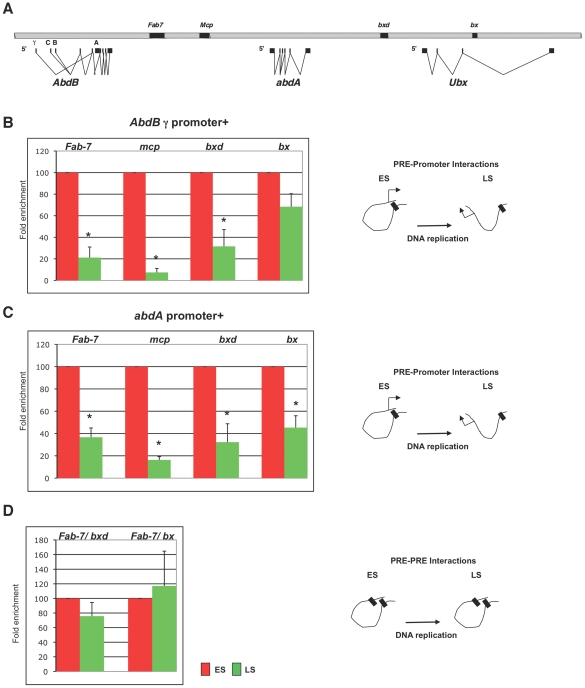
Higher order interactions are reduced during replication. (A) The scheme shows the Bithorax Complex (BX-C), including transcription units and genetically characterized regulatory regions. (B–D) Crosslinking frequencies observed in cells collected 1 h and 2 h from HU block release (ES, LS) are in red and in light green respectively. (B) Crosslinking frequencies, normalized on the ES fraction, between the *AbdB* γ promoter and PREs. (C) Crosslinking frequencies, normalized on the ES fraction, between the *abdA* promoter and PREs. (D) PRE/PRE crosslinking frequencies, normalized on the ES fraction. All data points were generated from an average of at least four independent experiments. Standard error of the mean is indicated. Two-tailed *t*-test was applied for statistical analysis. Asterisks indicate statistically relevant differences; α = 0.05. *P values*: *AbdB* γ promoter/*Fab-7: P* = 0.004; *AbdB* γ promoter/*Mcp*: *P* = 0.0001; *AbdB* γ promoter/*bxd*: *P* = 0.008; *AbdB* γ promoter/*bx*: *P* = 0.07; *abdA* promoter/*Fab-7*: *P* = 0.001; *abdA* promoter/*Mcp*: *P* = 0.0001; *abdA* promoter/*bxd*: *P* = 0.026; *abdA* promoter/*bx*: *P* = 0.004; *Fab-7*/*bxd*: *P* = 0.22; *Fab-7*/*bx*: *P* = 0.7.

We have previously demonstrated by Chromosome Conformation Capture (3C) and fluorescent *in situ* hybridization (FISH) that all major elements bound by PcG proteins, including PREs and core promoters, interact at a distance in the repressed state, resulting in a topologically complex structure necessary for the maintenance of BX-C silencing [16]. Similar results were obtained in mammals [24], [25]. In order to investigate whether PRE-mediated BX-C higher order structures are disrupted during replication, we used 3C analysis to monitor DNA/DNA interactions between PcG targets during S phase (Figure 2). We used synchronized cells collected 1 h and 2 h after the release from the HU block as representative of early and late S phase, respectively (Figure S1A). In comparing crosslinking frequencies of different fractions, we found that BX-C promoters were interacting with all PREs during early S phase, while during replication (late S phase) most PRE/promoters interactions were impaired (Figure 2B and 2C). These data may partially explain the previously reported dynamic nature of higher order interactions [13], [16] and suggest that during replication epigenetic higher order structures are altered and need to be reconstituted at each cell cycle. Interestingly, the frequencies of interaction between analysed PRE elements were stable throughtout DNA replication (Figure 2D), suggesting that PRE-PRE clustering may serve as the scaffolding template for PcG inheritance.

### PcG proteins and repressive mark H3K27me3 are enriched at PREs before replication

**Figure 3:**
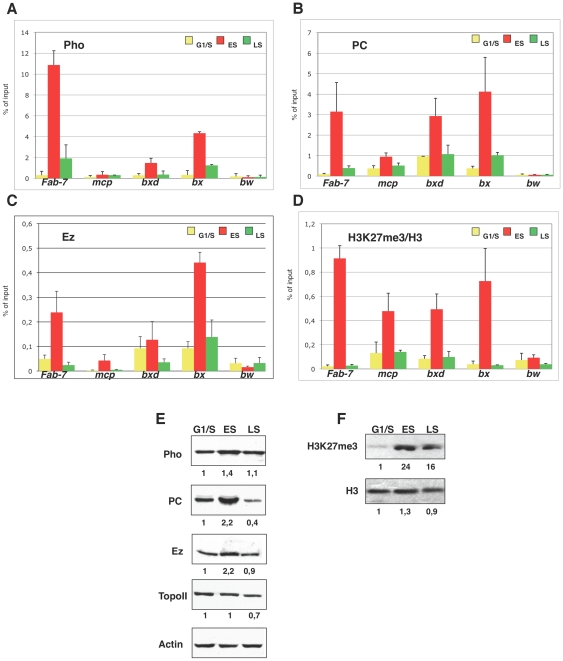
PcG proteins and repressive mark H3K27me3 are enriched at PREs before replication. (A–D) ChIP analysis are presented as percentage of input chromatin precipitated for each region. Mock enrichment is below 0.003% of the input. (A–C) ChIP analysis with antibodies against Pho, Pc and E(z) respectively on synchronized cells. Data obtained in HU treated cells (G1/S) are shown in yellow. Data obtained in cells collected 1 h and 2 h from HU block release (ES and LS) are in red and light green respectively. As negative control we used the promoter region of brown (*bw*) that is repressed in S2 but it is not under the control of PcG proteins [55]. Each graph shows the result from at least three independent immunoprecipitation reactions done on different chromatin preparations. Standard error of the mean is indicated. (D) ChIP enrichment for H3K27me3 normalized to histone H3 density. The graph shows the result from two independent immunoprecipitation reactions done on different chromatin preparations. Standard deviation is indicated. (E) Western blots of total protein extracts from synchronized S2 cells. Actin was used as a loading control. Quantifications of protein bands normalized on actin and relative to G1/S fraction are shown. (F) Western blots of histone extracts using antibodies that specifically recognize H3K27me3 and H3, as loading control. Quantifications of H3K27me3 and H3 bands relative to G1/S fraction are shown.

To dissect the dynamics of PcG proteins binding during S phase, we performed Chromatin Immunoprecipitation (ChIP) in synchronized S2 cells. Chromatin collected from G1/S, early and late S phase was immunoprecipitated with antibodies against PHO (Figure 3A), PC (Figure 3B) and E(Z) (Figure 3C), which are members of PhoRC, PRC1 and PRC2 complexes, respectively. Notably, all three PcG proteins were present at PREs during S phase, in agreement with and further corroborating previous reports [19]–[21]. However, we found that the amount of PcG proteins bound to target sites varied over S phase progression. In particular, we observed a striking increase, up to 10 fold, in early S phase (Figure 3A–3C), followed by a dramatic drop in PcG binding in late S phase, returning to G1/S basal levels. Thus PcG complexes engagement is uncoupled from and precedes target sites replication. To analyse PcG dependent HMTase function on chromatin we measured the levels of histone lysine methylation during S phase with antibodies that recognize total H3 and H3K27me3. Although total H3 levels at PREs did not change between G1/S and early S phase fractions (Figure S2A), the ratio between H3K27me3 and H3 peaked in early S phase (up to 10 fold; Figure 3D) following PcG protein loading onto PREs (Figure 3A–3C). Moreover, we observed a consistent drop of H3K27me3 from early to late S-phase while total H3 showed only a mild increase during PRE replication, suggesting that H3K27me3 trend during replication depends on mark deposition and not on replication dependent histone fluctuation. Little is known about *in vivo* dynamics of chromatin proteins during S-phase. Thus, as a further control, we looked at Topoisomerase II (TOPO II) an enzyme that plays a crucial role in DNA replication and binds PREs in *Drosophila* [26]. As shown in Figure S2B, we found that the amount of TOPO II at PREs did not change during S-phase, proving that the observed dynamic is specific for PcG complexes. As an additional control we performed ChIP experiments for repressive H3K9me3 mark that is also present on PREs [27], [28]. Interestingly, H3K9me3, also controlled by PcG proteins [28], showed a trend similar to H3K27me3 (Figure S2C) during S phase, suggesting that PcG epigenetic signatures are inherited at the same time during replication. Recently, phosphorylation of Serine 28 on histone H3 (H3S28ph) via mitogen and stress activated kinases, has been proposed as a novel mechanism that induces PcG chromatin displacement, counteracting the H3K27me3 docking site [29], [30]. To explore the contribution of the H3Ser28ph mark in S phase dependent PcG protein binding, additional ChIP experiments were performed. We found a progressive increase in H3Ser28ph mark from G1/S to late S phase (Figure S2D) both on PREs and the *bw* negative control, likely due to its role in mitosis [31]. Although we cannot completely exclude that H3K27me3 mark recognition by specific antibodies may be partially influenced by histone phosphorylation, the trend of H3Ser28ph mark did not correlate with the observed PcG and H3K27me3. This suggests that, at least during PRE replication, a putative signal dependent mechanism for PcG protein displacement by phosphorylation of Ser28 of histone H3 does not appear to be required.

Next we examined whether global levels of PcG proteins and H3K27me3 would be S phase regulated. Western blot and RT-PCR analysis revealed that both E(Z) and PC reached their maximum peak of expression during early S phase (Figure 3E and Figure S2E), while PHO showed only a slight increase. These dynamics were not observed for TOPO II, used as control (Figure 3E). The same trend was observed for total H3K27me3 while H3 levels remained constant throughout S phase (Figure 3F). We conclude that PcG proteins quantitatively engage their target sites and enrich for H3K27me3 epigenetic mark in early S phase, preceding PRE replication.

Despite the repression imposed on BX-C by PcG proteins in S2 cells it is possible to detect basal transcription levels of homeotic genes. To measure the correlation between the amount of PcG bound to its repressive function, we performed transcriptional analysis in synchronized S2 cells (Figure S3A). Different primer pairs were used to discriminate the mature and the primary transcripts of two homeotic genes, *Ubx* and *abdA*. We found a slight transcriptional increase in late S phase when Polycomb proteins are reduced on their targets (Figure S3A) and the analysed sequences are replicated (Figure S3B). However, we observed the same trend also with the late replicating *bw* negative control (Figure S3A and S3B), indicating that this effect is not dependent on PcG protein levels. We performed additional transcriptional analysis on synchronized cells treated with dsRNA against Pho, PC and Ez, which give rise to homeotic gene derepression (Figure S3C), and against Gfp as a control. The reproducible transcriptional trend found in PcG depleted cells lends support to the view that it is not dependent of PcG binding. These results suggest that, although continously repressed during S phase, some transcripts escape the restraint at the moment of DNA replication.

### Global levels of Polycomb protein and repressive mark H3K27me3 decrease from early to late S phase

**Figure 4:**
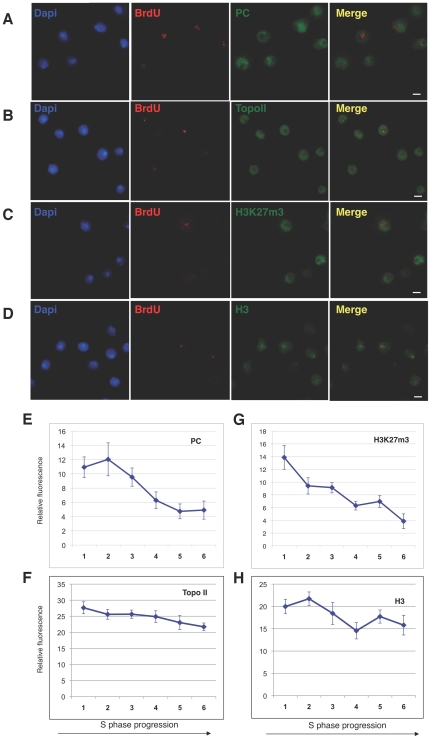
Relative Polycomb and H3K27me3 fluorescence change during S phase. (A–D) Representative examples of *D. melanogaster* S2 nuclei with double immunostaining using PC (A), Topoisomerase II (B), H3K27me3 (C) and H3 (D) with anti-BrdU antibodies. Scale bar = 10 µm. (E–H) Quantification of PC (E), Topoisomerase II (F), H3K27me3 (G) and H3 (H) intranuclear mean fluorescence intensity during S phase. Image stacks of 138, 208, 187 and 87 nuclei, respectively, with different expression levels were recorded using constant image acquisition parameters. All values are background corrected. Nuclei were grouped into six categories based on volumes measured in pixel units (1∶6000; 2∶ 7000; 3∶ 8000; 4∶9000; 5∶ 10000; 6∶11000). Mean PC, TOPO II, H3K27me3 or H3 relative fluorescence intensities were then determined for each category by averaging over all nuclei of a class. Standard error of the mean is indicated. One-way ANOVA was applied for statistical analysis. α = 0.05. *P values*: PC *p* = 0.001; H3K27me3 *p* = 0.0001.

As PcG proteins form discrete bodies in the nucleus [32], [33], we followed PC and H3K27me3 localization pattern during replication. To identify S phase, a S2 population of cells was pulse-labelled with BrdU and then analyzed by immunofluorescence (Figure 4A–4D and Figure S4A). As expected, PC does not colocalize with constitutive heterochromatin and it is excluded from replication foci (Figure 4A). This is in agreement with data in mammals showing no colocalization of the PRC1 subunit CBX8 with BrdU foci [19]. In order to perform a deeper analysis of PC dynamics, we then followed the time-lapse of the S phase by measuring nuclei dimensions. First, we used FACS to measure the mean cell size of two fractions representing early and late S phase (Figure 1A). We found that cells belonging to the early S phase were smaller compared with cells of the late S phase (Figure S4B), indicating that as S phase progresses, cell dimension increases. This allowed us to study protein distribution throughout S phase by immunofluorescence. Second, we quantified PC protein levels by measuring the intra-nuclear mean fluorescent intensity in BrdU positive cells. We plotted these values, classifying cells by dimension, and we found that PC amount decreased with S phase progression (Figure 4E). As a control, we repeated the experiment to study TOPO II dynamics and we found a more diffuse distribution of the protein in the cell (Figure 4B) and a slight decrease in overall levels in late S phase compared to early (Figure 4F), in agreement with Western blot results. In parallel, we followed the activity of PRC2 complex by analysing the distribution and the amount of H3K27me3 mark in BrdU positive cells. As observed for PRC1, we found that the repressive mark is excluded from constitutive heterochromatin and replication foci (Figure 4C) and that the relative nuclear intensity of H3K27me3 fluorescence decreased with a similar trend (Figure 4G). In H3 immunostaining, used as control, we found a strong signal in heterochromatin foci (Figure 4D) and relative nuclear fluorescence remained constant throughout S-phase (Figure 4H). Taken together, these data confirm results from ChIP and Western blot analysis, clearly showing specific dynamics of PC and histone H3K27me3 mark during replication.

### Early S phase dynamics of H3K27me3 repressive mark and EZH2 are conserved in human cells

**Figure 5:**
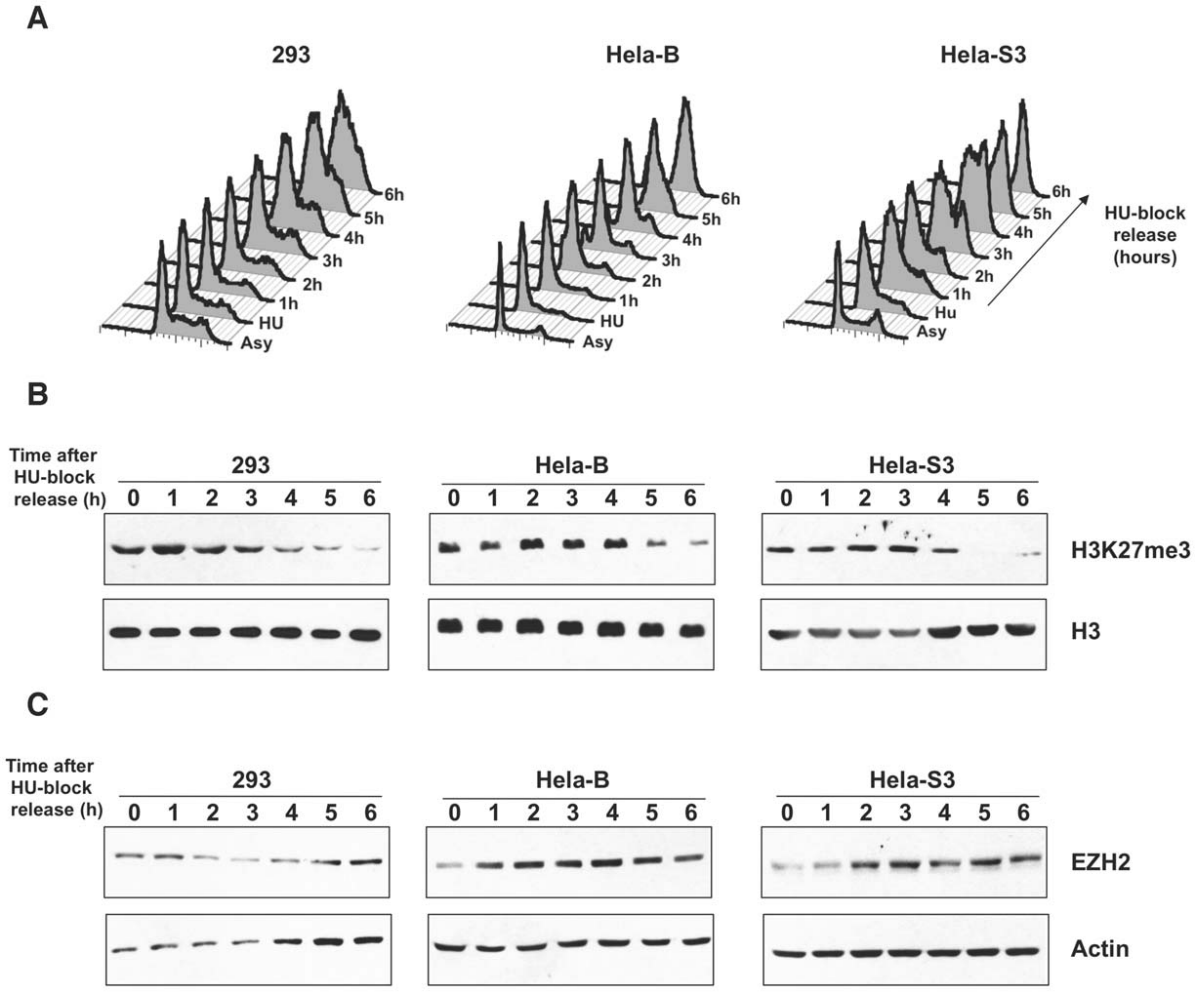
PcG and H3K27me3 fluctuations in S-phase are conserved. (A) Cell-cycle profile of human 293, Hela B and Hela S3 cells stained with propidium iodide before and after HU block release. Cells treated with HU are in G1/S phase. After HU block release, cells were collected at each hour, as indicated. (B) Western blots of histone extracts using antibodies that specifically recognize H3K27me3 and H3, as loading control. (C) Western blots of total protein extracts from synchronized cells. Actin was used as a loading control.

Previous reports indicated that PRC1 and 2 complexes are localized at silent *INK4/ARF* locus in proliferating mouse embryonic fibroblasts (MEF) and that this locus is replicated during late S phase [34]. This evidence was further supported by large scale analysis in Hela cells in which the presence of PcG H3K27me3 repressive mark positively correlated with late-replicating genomic regions, suggesting that PcG targets replicate during late S phase [35], [36]. To check if early S-phase PcG dynamics observed in *Drosophila* cells would be evolutionary conserved, we analysed the levels of H3K27me3 mark in synchronised mammalian cells. Three different human cell lines (293, Hela S3 and Hela B) were treated with HU and, after block release, several fractions from G1/S (HU block) to late S/G2 phase were collected (Figure 5A). H3K27me3 total amounts were measured by western blot analysis (Figure 5B). Strikingly, all three cellular systems revealed a trend similar to *Drosophila* S2 cells, showing H3K27me3 enrichment in the first part of the S phase and then a gradual reduction along with S phase progression. Interestingly, it has been previously shown that Hela cells collected 6 hours after G1/S block release can be considered representative of late replication [35], indicating that H3K27me3 deposition precedes duplication of potentially silenced, late replicating sequences. In the same samples, to correlate the H3K27me3 mark deposition with PcG dependent HMTase function on chromatin we analysed the human homologue of *Drosophila* Ez protein, EZH2 (Figure 5C). As for *Drosophila*, western blot analysis indicate that increase in H3K27me3 was accompanied by increase in EZH2 levels. These findings suggest that a characteristic timing of PRC2 activity and H3K27me3 deposition preceding PcG target replication may be a key and evolutionary conserved mechanism for epigenetic inheritance of gene silencing. Interestingly, the H3K27me3 drop in late S phase was not followed by a comparable decrease of EZH2 levels, suggesting that other cell-cycle coupled mechanisms could be involved in EZH2 HMT activity regulation.

## Discussion

At each cell cycle, the integrity of genetic and epigenetic information is challenged during DNA replication, when chromatin undergoes a wave of disruption and subsequent restoration in the wake of the passage of the replication machinery. It is well described that assembly of core histones is coincident with DNA replication and takes place at the replication fork [37]–[41]. However, temporal re-establishment of epigenome structure during cell division remains a key question in epigenetic research.

In mammals, mechanisms of heterochromatin formation involve the sequential recruitment of HMT, deposition of histone mark and binding of HP1 chromodomain protein at the replication fork [42]. In yeast, generation of short interfering RNAs from centromeric repeats in S-phase allows the loading of heterochromatin factors that, in turn, restore the H3K9me2 mark after replication [43], [44]. Despite the considerable amount of information about PcG catalytic and repressive functions, by now mechanisms of PcG mediated epigenetic inheritance at cell division are not fully understood. Increasing evidences suggest that PcG mediated epigenetic signatures are cell cycle regulated, being controlled in S and M phases, when cells are subjected to profound modifications of chromosomal components and nuclear structure [45]–[48].

The key question, still open, is how PcG dependent epigenetic marks are inherited when the genome is replicated. Studies in mammalian cells suggest that all three proteins of PRC2 in the trimeric complex are required to form a combined binding surface that can recognize the H3K27me3 modification, thus generating a positive feedback loop that helps to propagate H3K27me3 mark through DNA replication. Since also PRC1 can recognize H3K27me3, via chromodomain proteins, this could also be a mechanism for recruiting new PRC1 complexes following DNA replication [19], [20]. Notably, it has been shown that in *Drosophila* chromatin histone proteins turn over faster than cell cycle suggesting that in principle they may be loaded on DNA not only during S phase [49]. Indeed, the ability of PcG proteins to bind their own mark, occurring during all phases of cell cycle and reinforcing the epigenetic repressed status of target genes, could partially explain the stability of epigenetic signatures despite their high turnover in the cell [19], [20]. On other hand, during replication, stability of epigenetic marks is challenged by the replication fork passage. Hence, specific mechanisms of epigenetic inheritance in S-phase must be provided in order to preserve cell identity.

We addressed this issue by analyzing the *in vivo*, cell cycle dependent dynamics of PcG proteins and their role in maintaining BX-C homeotic gene silencing. By using different experimental approaches, we show that components of the three major PcG complexes follows a characteristc dynamics in S-phase and, notably, it is uncoupled from replication timing. We found that, in early S phase, endogenous PcG protein levels increase and PcG complexes chromatin loading and enrichment for characteristic H3K27me3 mark are strongly enhanced (Figure 3). All these events precede late S-phase when PREs are replicated (Figure 1) and most of epigenetic signatures, such as looping between regulatory sequences (Figure 2), PcG binding and H3K27me3 mark levels (Figure 3 and Figure 4), appear to be challenged. These results are supported by experiments on synchronized mammalian cells showing a conserved dynamics of PcG proteins and H3K27me3 mark through S phase (Figure 5). Of note, our data are in line with previous findings based on mass spectrometry quantification on parental versus newly deposited histones showing that the establishment of H3K27me3 patterns during cell cycle takes place to large extent before replication [47]. Further, the conclusions that can be drawn from these data are strongly reminiscent of centromeric heterochromatin duplication in which epigenetic inheritance of histone variant CENP-A (centromeric protein A) is restricted to a brief interval in G1 and subsequent dilution occurs during S phase [50], [51]. Thus, we propose a mechanism for PcG epigenetic signature inheritance in which H3K27me3 mark is actually inherited by dilution and not by *de novo* methylation occurring at the time of replication.

Overexpression of PcG proteins and consequent changes in specific chromatin landscapes have been extensively documented in human cancer [52], where control on the cell cycle is lost, and cells constantly enter S phase. We suggest that higher levels of PcG proteins characteristic of cancer cells might be needed to maintain transcriptional repression on differentiation genes and oncosuppressors through S phase. The identification of PcG regulated epigenetic inheritance time window may be relevant for cell reprogramming by allowing the modulation of cell memory function [48], [53].

## Materials and Methods

### Culture cell growth

*Drosophila* embryonic S2 cells were grown at 25°C in serum-free insect culture medium (HyQ SFX; Hyclone, Logan, UT). 293, Hela B, and Hela S3 were cultured in Dulbecco Modified Eagel's Medium (DMEM) supplemented with penicillin/streptomycin and 10% fetal bovine serum (Euroclone).

### Replication timing analysis

Exponentially growing S2 cells (1×10^6^ cells/ml) were cultured in presence of 50 µM Bromodeoxyuridine (BrdU) for 60 min. For sorting, cells were divided into aliquots containing 5×10^6^ cells per tube, washed with cold PBS, resuspended in 0.5 ml of cold PBS, fixed with drop by drop addition of 5 ml of 70% cold ethanol and incubated for 1 h on ice. Cells were then washed with PBS, resuspended in PBS/RNase A (1 mg/ml) 30 min at 37°C followed by addition of Propidium Iodide (20 µg/ml) and incubated 30 min in the dark at 4°C. On the basis of DNA content, cells were sorted into different S phase fractions using two selective gates representing roughly the first and the last thirds of S phase. Equal numbers of cells from each cell cycle fraction (150,000) were sorted (using a Becton Dickinson or a Moflo, Coulter) into microcentrifuge tubes containing lysis buffer (50 mM TrisHCl pH 8; 10 mM EDTA; 0,8% SDS; supplemented with 0.2 mg of proteinase K per ml). For analysis after HU synchronization, cells representing early S phase were pulse-labelled with BrdU at the beginning of S phase and collected 1 h from HU block release, while cells representing late S phase were pulse labelled with BrdU after 1 h and collected after 2 h from HU block release and resuspended in lysis buffer. The aliquots, collected either by FACS or after synchronization, were incubated at 50°C for 2 h in lysis buffer and then stored at −20°C. Lysates were then extracted once with phenol-chloroform, and phenol was extracted again with an additional volume of TE1X. DNA was precipitated with sodium acetate and ethanol and resuspended in 500 µl of TE. DNA was sonicated to an average size of 0.5 kb, and an aliquot of 100 µl was checked on agarose gel. DNA was heat denatured 10 min at 95°C and cooled on ice. Then 50 µl of 10× phosphate buffer (1 M Sodium phosphate [pH 7.0], 1.4 M NaCl; 0.5% Triton X-100) and 40 µl of mouse anti-BrdU DNA monoclonal antibody (25 mg/ml Becton Dickinson) were added to each tube. After 2 h of constant rocking at room temperature, protein AG plus agarose beads (Santa Cruz Biotechnology) were added and incubation continued for an additional hour at room temperature with rocking. DNA-protein complexes were pelleted by microcentrifuging for 5 min at 4°C. After washing with 750 µl of 1× phosphate buffer, pellets were resuspended in 200 µl of digestion buffer (50 mM Tris HCl pH 8, 10 mM EDTA, 0,5% SDS, 250 µg/ml proteinase K). Digestion was allowed to proceed overnight at 37°C and then for 1 h at 50°C after the addition of 100 µl of fresh digestion buffer. DNA was extracted and precipitated as above, briefly dried and resuspended in 40 µl of TE. RT PCR was performed using 1 µl of each nascent strand sample as template. Primer sequences: CG10873-f 5′agcttgctgcgcagcgag3′, CG10873-r 5′tctccaggcagaagactaagg3′; dodeca-f 5′actggtcgcgtactggtcc3′, dodeca-r 5′gtctcgtactctgtcccgtatt3′; fab-7-f 5′gaaaatgcccaacaaaatgc3′, fab-7-r 5′cgctgtctcgcctcttcttc3′; mcp-f 5′tgcggacgccatttgacac3′, mcp-r 5′gagccacgcagcgagttc3′; bxd-f 5′tcgtcgcttgtttggataattact3′, bxd-r 5′tgcggtgataaggtccataatc3′; bx-f 5′ttattgttgctacaccgctg3′, bx-r 5′agtaggtgccgcgtatgtg3′; CG3436–f 5′atcgctaacagccatgtcgg3′, CG3436-r 5′cttaccgattcaaggagcgc3′; CG4345-f 5′ttcccgagtctctcaccgc3′, CG4345-r 5′acaggaacccacaccactgac3′; Ubxpr-f 5′tcagccctcctccatgatg3′, Ubxpr-r 5′ccaaatcgcagttgccagtg3′; abdApr-f 5′ttgagtcagggagtgagcc3′, abdApr-r 5′cgctttgagtcgttggagac3′; bwpr-f 5′tgatgagcgacaattagctgg3′, bwpr-r 5′tgtccgtctgtctgtctgtc3′.

### Chromosome conformation capture (3C)

The 3C assay was performed as previously described [16].

### Antibodies

Antibodies against PC were kindly provided by R. Paro, antibodies against Topoisomerase II by D. Arndt-Jovin, and antibodies against Pho and E(z) by J. Muller. Commercial rabbit polyclonal antibodies against methylated Lysine 27 of histone H3 (Upstate, 07-449), methylated Lysine 9 of histone H3 (Abcam, ab8898), phosphorilated Serine 28 of histone H3 (Upstate, 07-145), histone H3 (Abcam, ab1791) and EZH2 (Diagenode pAb-039-050) were used.

### Chromatin immunoprecipitation

ChIP experiments were performed as previously described [27] with minor modifications. After synchronization, cells of different phases of cell cycle were fixed in 1% formaldehyde for 15 min at room temperature and quenched by addition of glycine at 125 mM final concentration for 5 min at room temperature before being placed on ice. Cells were washed once with ice-cold PBS, resuspended in ice-cold cell lysis buffer (5 mM Pipes pH 8; 85 mM KCl; 0.5% NP40; 1 mM PMSF; 1× Protease Inhibitors) and left on ice for 10 min. After centrifugation at 2000 rpm for 5 min, nuclei were resuspended in ice-cold nuclear lysis buffer (50 mM TrisHCl pH 8.0; 10 mM EDTA; 0.8% SDS; 1 mM PMSF; 1× Protease Inhibitors) and left for 10 min on ice. Chromatin was sonicated in the presence of glass beads (150–200 mm, Sigma), spun for 10 min at maximum speed at 4°C, diluted to 0.2% SDS with dilution buffer (10 mM Tris–HCl pH 8.0, 0.5 mM EGTA, 1% Triton X-100, 140 mM NaCl), then split into aliquots and processed immediately for IP. For pre-clearing and antibody recovery, Protein A/G Plus-agarose beads (Santa Cruz Biotechnology) were used. After washing, samples and control chromatin (input) were incubated in the presence of 2 µl of Rnase cocktail (DNase-free, Ambion) overnight at 65°C. Then, samples were adjusted to 0.5% SDS and 0.5 mg/ml proteinase K and incubated for additional 2 h at 55°C. The DNA was phenol–chloroform extracted and precipitated. The final pellet was resuspended in 30 µl of TE and stored at 4°C for RT-PCR analysis. Primer sequences are indicated above.

### Protein and histone extraction

Total proteins were prepared by resuspending 2×10^6^ S2 or 1×10^6^ mammalian cells in extraction buffer (50 mM TrisHCl pH 7.6; 0.15 M NaCl; 5 mM EDTA; 1× Protease Inhibitors; 1% Triton X-100). Three pulses of 10 sec sonication at 30% amplitude were performed to allow dissociation of protein from chromatin and solubilization.

For histone extraction, 8×10^6^ S2 cells were washed in cold 1× PBS and resuspended in 800 µl of extraction buffer (10 mM Hepes pH 8; 0.1 mM MgCl_2_; 0.1 mM EDTA; 2 mM PMSF; 1× Protease Inhibitors; 1 mM NaF; 1 mM Na_3_VO_4_) and passed through a needle on ice. After incubation of 10 min on ice, cells were centrifuged for 10 min at 4°C at 2000 rpm. Pellets were washed with 400 µl of extraction buffer, resuspended in 100 µl of 0.2 N HCl and incubated overnight at 4°C with constant rocking. After 10 min of centrifugation at 13000 rpm 4°C, the supernatant was run on 12% SDS-PAGE. Alternatively, 30 µg of mammalian or *Drosophila* protein extracts were treated for 1 h with 4 units of DNAse (Turbo DNAse Ambion) at 37°C. For Western blot analysis, the densities of protein bands were measured using Image J software program.

### Real-time PCR analysis

Total RNA was isolated with Trizol reagent (Invitrogen). 1 µg of RNA from each sample was subjected to cDNA synthesis using a Quantitect reverse transcription kit (Qiagen). DNA from ChIP, 3C or cDNA preparation was amplified in 20 µl reaction mixtures in the presence of 10 µl 2× QuantiTect SYBR Green master mix (Qiagen) and 0.5 µM of corresponding primers. Real-time PCR was performed with the DNA Engine Opticon 2 (MJ). Copy number was determined using the cross-point (Cp) value, which is automatically calculated using the Opticon Monitor 2 software (MJ). Primer sequences: rtgapdh-f 5′aagggaatcctgggctacac3′, rtgapdh-r 5′accgaactcgttgtcgtacc3′; rtpho-f 5′tcagttggttcacaccggtg3′, rtpho-r 5′gaggtatcttcactctggctg3′; rtpc-f 5′ttcaagactcaagtgctgcc3′, rtpc-r 5′ccatgggaaataagcaggag3′; rtez-f 5′ctgtggctgagatcaactcc3′, rtez-r 5′gacaggtcttggtcagcatg3′; rtbw-f 5′tcgctgtgcctcgagtgg3′, rtbw-r 5′aatcgccgccagcagcg3′; rtUbx-f 5′agtgtcagcggcggcaac3′, rtUbx-r 5′agtctggtagaagtgagcccg3′; rtabdA-f 5′caaatacaacgcaacccgagac3′, rtabdA-r 5′agcgatcgtgttgctgctg3′; utrgapdh-f 5′cgaactgaaactgaacgagag3′, utrgapdh-r 5′ttgacatcgatgaagggatcg3′; utrUbx-f 5′gttcgatggcaacggattgg3′, utrUbx-r 5′tgacggatttcctcgaatctg3′; utrabdA-f 5′aactcactgtgtgcggttcg3′, utrabdA-r 5′tcaagtgcgtgagtgtgtgtg3′; utrbw-f 5′agtcggcacatcacatagcc3′, utrbw-r 5′gttccagaaactgtagttgctc3′.

### Immunostainings

For BrdU labelling, exponential S2 cells were grown for one hour in the presence of 50 µM BrdU. 10^6^ cells were centrifuged, resuspended in 0.4 ml of medium and placed at room temperature (RT) for 30 min on a Poly-Lysine coated slide (22 mm×22 mm). Fixation was performed in 4% paraformaldehyde 1× PBS for 10 min at RT. Cells were washed 3 times with PBT (PBS 1×, 0.1% Tween 20), incubated for 1 h at RT with RNAseA (100 µg/ml in PBT) and for 10 min at RT with PBS, 0.5% Triton. After washing cells again in PBS, they were incubated for 2 min at RT in 0.07N NaOH, briefly rinsed twice in PBS and blocked in PBS/1%BSA. All antibody hybridizations were carried out in a humid atmosphere at 37°C. Anti-PC and anti-H3K27me3 antibodies were incubated for 12–16 h while anti-BrdU antibody was incubated for 1 h. Washes were done in PBT. DNA was counterstained with DAPI, and glasses were mounted in Vectashield Antifade (Vector Laboratories). Images were taken with a Nikon ECLIPSE 90i microscope (100× objective) that was equipped with a digital camera (Nikon Coolpix 990) and NIS-Element software. Fluorescence quantification was done by determining the intranuclear mean fluorescence intensity using an Image J software program that computes area, mean, and grey values.

### RNAi

Exonic fragments of 600 bp, 1400 bp, 658 bp or 810 bp, respectively, from *Gfp*, *Pc*, *Pho* or *E(z)* genes, were amplified by PCR, creating T7 polymerase binding sites for the transcription of both strands. RNAi was performed as described previously [54]. Primer sequences: *Gfp* 5′acgtaaacggccacaagttc3′-5′tgctcaggtagtggttgtcg3′; *Pc* 5′attggcaagttaagcacgggca3′-5′acatcctggatcgccgcctca3′; *Pho* 5′acagtacgatgaagatataggc3′-5′tgatctgaactgagcttatagg3′; *E(z)* 5′tcgaaggcattatgaatagcac3′-5′atccgcatcttcagtctcc3′.

## Supporting Information

Figure S1 Experimental strategy to measure the timing of DNA replication using HU synchronization (A) Cell-cycle profile of *D. melanogaster* S2 cells stained with propidium iodide before and after HU block release. Cells treated with HU are in G1/S phase. After 1 h from the HU block release, cells are considered in early S phase, while cells collected 2 h from the release are in late S phase. (B) Replication timing of PREs as measured by Real Time PCR (qRTPCR). Ratios between the amplified products in early and late S phase are shown. We amplified positive controls for the early and late S phase and gene names correspond to their entries in FlyBase. All data points were generated from an average of four independent experiments. Standard error of the mean is indicated. Two-tailed *t*-test was applied for statistical analysis. Asterisks indicate statistically relevant differences; α = 0.05. *P values*: 108735/dodeca: *P* = 0.033; 108735/*Fab-7: P* = 0.013; 108735/*mcp: P* = 0.011; 108735/*bxd: P* = 0.006; 108735/*bx: P* = 0.006.
(TIFF)

Figure S2 PcG dependent repressive mark are enriched at PREs before replication. (A–D) ChIP analysis with antibodies against H3 (A), Topoisomerase II (B), H3K9me3 (C) and H3S28ph (D) on synchronized cells. ChIP analysis are presented as percentage of input chromatin precipitated for each region. Mock enrichment is below 0.003% of the input. ChIP enrichment for H3 modifications are normalized to histone H3 density. Data obtained in HU treated cells (G1/S) are shown in yellow. Data obtained in cells collected 1 h and 2 h from HU block release (ES and LS) are in red and light green respectively. Each graph shows the result from at least three independent immunoprecipitation reactions done on different chromatin preparations. Standard error of the mean is indicated. (E) Quantification of transcription by qRTPCR. The transcription levels of PcG mRNA are shown as percentage of *Gapdh* expression. All data points were generated from the results of six independent experiments. Standard error of the mean is indicated.
(TIFF)

Figure S3 Transcriptional profile of homeotic genes during S phase progression is not affected by PcG proteins depletions. (A) Quantification of transcription levels of mature and primary transcripts of indicated genes by Real Time PCR in untreated S2 and Gfp-dsRNA, Pc-dsRNA, Pho-dsRNA or Ez-dsRNA treated S2 cells. Data obtained in HU blocked cells (G1/S) are shown in yellow. Data obtained in cells collected 1 h and 2 h from HU block release (ES and LS) are in red and light green respectively. Transcription levels are shown as percentage of *Gapdh* expression. No amplification was detected in the absence of RT. All data points were generated from the results of at least four independent experiments. Standard error of the mean is indicated. (B) Replication timing of analysed promoters as measured by qRTPCR. Ratios between the amplified products in early and late S phase are shown. We amplified positive controls for the early and late S phase and gene names correspond to their entries in FlyBase. All data points were generated from an average of at least three independent experiments. Standard error of the mean is indicated. Two-tailed *t*-test was applied for statistical analysis. Asterisks indicate statistically relevant differences; α = 0.05. *P values*: CG3436/CG4345: *P* = 0.0002; CG3436/*Ubx promoter: P* = 0.003; CG3436/*abdA promoter: P* = 0.004; CG3436/*bw promoter: P* = 0.01. (C) Quantification of transcripts by qRTPCR. Expression level of homeotic genes in GFP-RNAi S2 cells (blue), in PHO-dsRNA treated cells relative to GFP-RNAi S2 (violet), in PC-dsRNA treated cells relative to GFP-RNAi S2 (brown), in Ez-dsRNA treated cells relative to GFP-RNAi S2 (green). Transcriptional levels are shown as percentage of *Gadph* expression. All data points were generated from an average of four different experiments. Standard error of the mean is indicated.
(TIFF)

Figure S4 (A) Negative control of immunofluorescence experiment. Representative examples of S2 nuclei with double immunostaining using only secondary antibodies. (B) Cell dimensions in S phase measured by FACS. Dot plot indicating the mean FSC (Forward Scatter) of early (ES, red) and late S (LS, green) phase in 5 independent S2 populations. *Student t test* was applied for statistical analysis; α = 0.05. *P* = 0.0009.
(TIFF)

## References

[1] Morey L, Helin K (2010). Polycomb group protein-mediated repression of transcription.. Trends Biochem Sci.

[2] Fritsch C, Brown JL, Kassis JA, Muller J (1999). The DNA-binding polycomb group protein pleiohomeotic mediates silencing of a Drosophila homeotic gene.. Development.

[3] Mihaly J, Mishra RK, Karch F (1998). A conserved sequence motif in Polycomb-response elements.. Mol Cell.

[4] Wang L, Brown JL, Cao R, Zhang Y, Kassis JA (2004). Hierarchical recruitment of polycomb group silencing complexes.. Mol Cell.

[5] Cao R, Wang L, Wang H, Xia L, Erdjument-Bromage H (2002). Role of histone H3 lysine 27 methylation in Polycomb-group silencing.. Science.

[6] Czermin B, Melfi R, McCabe D, Seitz V, Imhof A (2002). Drosophila enhancer of Zeste/ESC complexes have a histone H3 methyltransferase activity that marks chromosomal Polycomb sites.. Cell.

[7] Kuzmichev A, Nishioka K, Erdjument-Bromage H, Tempst P, Reinberg D (2002). Histone methyltransferase activity associated with a human multiprotein complex containing the Enhancer of Zeste protein.. Genes Dev.

[8] Muller J, Hart CM, Francis NJ, Vargas ML, Sengupta A (2002). Histone methyltransferase activity of a Drosophila Polycomb group repressor complex.. Cell.

[9] Francis NJ, Kingston RE, Woodcock CL (2004). Chromatin compaction by a polycomb group protein complex.. Science.

[10] Wang H, Wang L, Erdjument-Bromage H, Vidal M, Tempst P (2004). Role of histone H2A ubiquitination in Polycomb silencing.. Nature.

[11] Scheuermann JC, de Ayala Alonso AG, Oktaba K, Ly-Hartig N, McGinty RK (2010). Histone H2A deubiquitinase activity of the Polycomb repressive complex PR-DUB.. Nature.

[12] Bantignies F, Grimaud C, Lavrov S, Gabut M, Cavalli G (2003). Inheritance of Polycomb-dependent chromosomal interactions in Drosophila.. Genes Dev.

[13] Bantignies F, Roure V, Comet I, Leblanc B, Schuettengruber B (2011). Polycomb-Dependent Regulatory Contacts between Distant Hox Loci in Drosophila.. Cell.

[14] Cleard F, Moshkin Y, Karch F, Maeda RK (2006). Probing long-distance regulatory interactions in the Drosophila melanogaster bithorax complex using Dam identification.. Nat Genet.

[15] Comet I, Savitskaya E, Schuettengruber B, Negre N, Lavrov S (2006). PRE-mediated bypass of two Su(Hw) insulators targets PcG proteins to a downstream promoter.. Dev Cell.

[16] Lanzuolo C, Roure V, Dekker J, Bantignies F, Orlando V (2007). Polycomb response elements mediate the formation of chromosome higher-order structures in the bithorax complex.. Nat Cell Biol.

[17] Sing A, Pannell D, Karaiskakis A, Sturgeon K, Djabali M (2009). A vertebrate Polycomb response element governs segmentation of the posterior hindbrain.. Cell.

[18] Woo CJ, Kharchenko PV, Daheron L, Park PJ, Kingston RE (2010). A region of the human HOXD cluster that confers polycomb-group responsiveness.. Cell.

[19] Hansen KH, Bracken AP, Pasini D, Dietrich N, Gehani SS (2008). A model for transmission of the H3K27me3 epigenetic mark.. Nat Cell Biol.

[20] Margueron R, Justin N, Ohno K, Sharpe ML, Son J (2009). Role of the polycomb protein EED in the propagation of repressive histone marks.. Nature.

[21] Francis NJ, Follmer NE, Simon MD, Aghia G, Butler JD (2009). Polycomb proteins remain bound to chromatin and DNA during DNA replication in vitro.. Cell.

[22] Schubeler D, Scalzo D, Kooperberg C, van Steensel B, Delrow J (2002). Genome-wide DNA replication profile for Drosophila melanogaster: a link between transcription and replication timing.. Nat Genet.

[23] Schwaiger M, Stadler MB, Bell O, Kohler H, Oakeley EJ (2009). Chromatin state marks cell-type- and gender-specific replication of the Drosophila genome.. Genes Dev.

[24] Kheradmand Kia S, Solaimani Kartalaei P, Farahbakhshian E, Pourfarzad F, von Lindern M (2009). EZH2-dependent chromatin looping controls INK4a and INK4b, but not ARF, during human progenitor cell differentiation and cellular senescence.. Epigenetics & Chromatin.

[25] Tiwari VK, McGarvey KM, Licchesi JD, Ohm JE, Herman JG (2008). PcG proteins, DNA methylation, and gene repression by chromatin looping.. PLoS Biol.

[26] Lupo R, Breiling A, Bianchi ME, Orlando V (2001). Drosophila chromosome condensation proteins Topoisomerase II and Barren colocalize with Polycomb and maintain Fab-7 PRE silencing.. Mol Cell.

[27] Breiling A, O'Neill LP, D'Eliseo D, Turner BM, Orlando V (2004). Epigenome changes in active and inactive polycomb-group-controlled regions.. EMBO Rep.

[28] Papp B, Muller J (2006). Histone trimethylation and the maintenance of transcriptional ON and OFF states by trxG and PcG proteins.. Genes Dev.

[29] Gehani SS, Agrawal-Singh S, Dietrich N, Christophersen NS, Helin K (2010). Polycomb group protein displacement and gene activation through MSK-dependent H3K27me3S28 phosphorylation.. Mol Cell.

[30] Lau PN, Cheung P (2011). Histone code pathway involving H3 S28 phosphorylation and K27 acetylation activates transcription and antagonizes polycomb silencing.. Proc Natl Acad Sci U S A.

[31] Perez-Cadahia B, Drobic B, Davie JR (2009). H3 phosphorylation: dual role in mitosis and interphase.. Biochem Cell Biol.

[32] Buchenau P, Hodgson J, Strutt H, Arndt-Jovin DJ (1998). The distribution of polycomb-group proteins during cell division and development in Drosophila embryos: impact on models for silencing.. J Cell Biol.

[33] Ficz G, Heintzmann R, Arndt-Jovin DJ (2005). Polycomb group protein complexes exchange rapidly in living Drosophila.. Development.

[34] Agherbi H, Gaussmann-Wenger A, Verthuy C, Chasson L, Serrano M (2009). Polycomb mediated epigenetic silencing and replication timing at the INK4a/ARF locus during senescence.. PLoS ONE.

[35] Birney E, Stamatoyannopoulos JA, Dutta A, Guigo R, Gingeras TR (2007). Identification and analysis of functional elements in 1% of the human genome by the ENCODE pilot project.. Nature.

[36] Thurman RE, Day N, Noble WS, Stamatoyannopoulos JA (2007). Identification of higher-order functional domains in the human ENCODE regions.. Genome Res.

[37] English CM, Adkins MW, Carson JJ, Churchill ME, Tyler JK (2006). Structural basis for the histone chaperone activity of Asf1.. Cell.

[38] English CM, Maluf NK, Tripet B, Churchill ME, Tyler JK (2005). ASF1 binds to a heterodimer of histones H3 and H4: a two-step mechanism for the assembly of the H3-H4 heterotetramer on DNA.. Biochemistry.

[39] Groth A, Corpet A, Cook AJ, Roche D, Bartek J (2007). Regulation of replication fork progression through histone supply and demand.. Science.

[40] Groth A, Rocha W, Verreault A, Almouzni G (2007). Chromatin challenges during DNA replication and repair.. Cell.

[41] Natsume R, Eitoku M, Akai Y, Sano N, Horikoshi M (2007). Structure and function of the histone chaperone CIA/ASF1 complexed with histones H3 and H4.. Nature.

[42] Quivy JP, Roche D, Kirschner D, Tagami H, Nakatani Y (2004). A CAF-1 dependent pool of HP1 during heterochromatin duplication.. Embo J.

[43] Chen ES, Zhang K, Nicolas E, Cam HP, Zofall M (2008). Cell cycle control of centromeric repeat transcription and heterochromatin assembly.. Nature.

[44] Kloc A, Zaratiegui M, Nora E, Martienssen R (2008). RNA interference guides histone modification during the S phase of chromosomal replication.. Curr Biol.

[45] Chen S, Bohrer LR, Rai AN, Pan Y, Gan L (2010). Cyclin-dependent kinases regulate epigenetic gene silencing through phosphorylation of EZH2.. Nat Cell Biol.

[46] Kaneko S, Li G, Son J, Xu CF, Margueron R (2010). Phosphorylation of the PRC2 component Ezh2 is cell cycle-regulated and up-regulates its binding to ncRNA.. Genes Dev.

[47] Scharf AN, Barth TK, Imhof A (2009). Establishment of histone modifications after chromatin assembly.. Nucleic Acids Res.

[48] Wei Y, Chen YH, Li LY, Lang J, Yeh SP (2011). CDK1-dependent phosphorylation of EZH2 suppresses methylation of H3K27 and promotes osteogenic differentiation of human mesenchymal stem cells.. Nat Cell Biol.

[49] Deal RB, Henikoff JG, Henikoff S (2010). Genome-wide kinetics of nucleosome turnover determined by metabolic labeling of histones.. Science.

[50] Henikoff S, Furuyama T, Ahmad K (2004). Histone variants, nucleosome assembly and epigenetic inheritance.. Trends Genet.

[51] Loyola A, Almouzni G (2007). Marking histone H3 variants: how, when and why?. Trends Biochem Sci.

[52] Mills AA (2010). Throwing the cancer switch: reciprocal roles of polycomb and trithorax proteins.. Nat Rev Cancer.

[53] Sawarkar R, Paro R (2010). Interpretation of developmental signaling at chromatin: the Polycomb perspective.. Dev Cell.

[54] Breiling A, Turner BM, Bianchi ME, Orlando V (2001). General transcription factors bind promoters repressed by Polycomb group proteins.. Nature.

[55] Paro R, Zink B (1993). The Polycomb gene is differentially regulated during oogenesis and embryogenesis of Drosophila melanogaster.. Mech Dev.

