# Supplementary figures and images for: PcG Complexes Set the Stage for Epigenetic Inheritance of Gene Silencing in Early S Phase before Replication
Source: PLoS Genet. 2011 Nov 3;7(11):e1002370. doi: 10.1371/journal.pgen.1002370 (PMC3207895; doi:10.1371/journal.pgen.1002370)

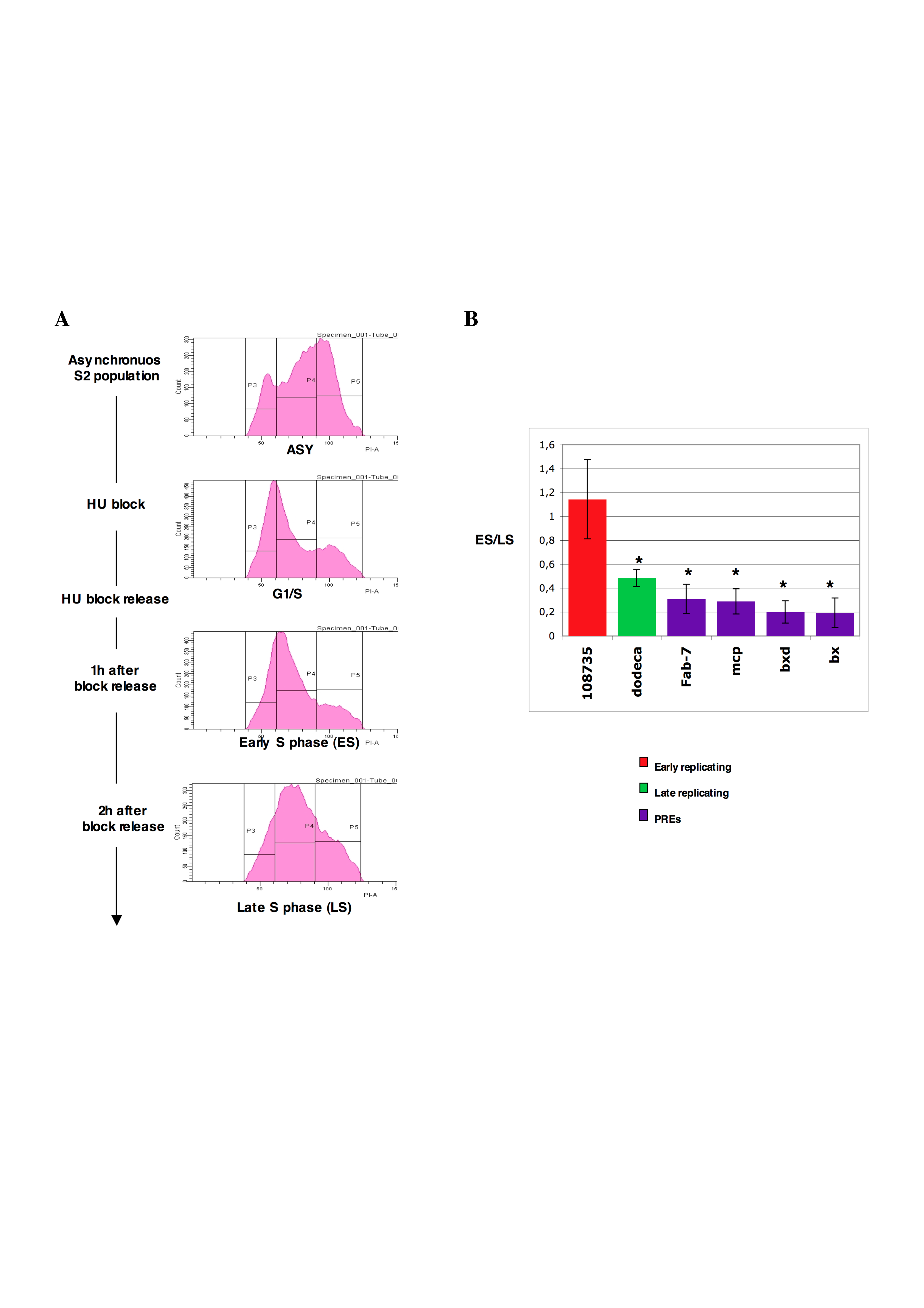

Supplement: Figure S1 — Experimental strategy to measure the timing of DNA replication using HU synchronization (A) Cell-cycle profile of D. melanogaster S2 cells stained with propidium iodide before and after HU block release. Cells treated with HU are in G1/S phase. After 1 h from the HU block release, cells are considered in early S phase, while cells collected 2 h from the release are in late S phase. (B) Replication timing of PREs as measured by Real Time PCR (qRTPCR). Ratios between the amplified products in early and late S phase are shown. We amplified positive controls for the early and late S phase and gene names correspond to their entries in FlyBase. All data points were generated from an average of four independent experiments. Standard error of the mean is indicated. Two-tailed t-test was applied for statistical analysis. Asterisks indicate statistically relevant differences; α = 0.05. P values: 108735/dodeca: P = 0.033; 108735/Fab-7: P = 0.013; 108735/mcp: P = 0.011; 108735/bxd: P = 0.006; 108735/bx: P = 0.006. (TIFF) [file pgen.1002370.s001.tiff]

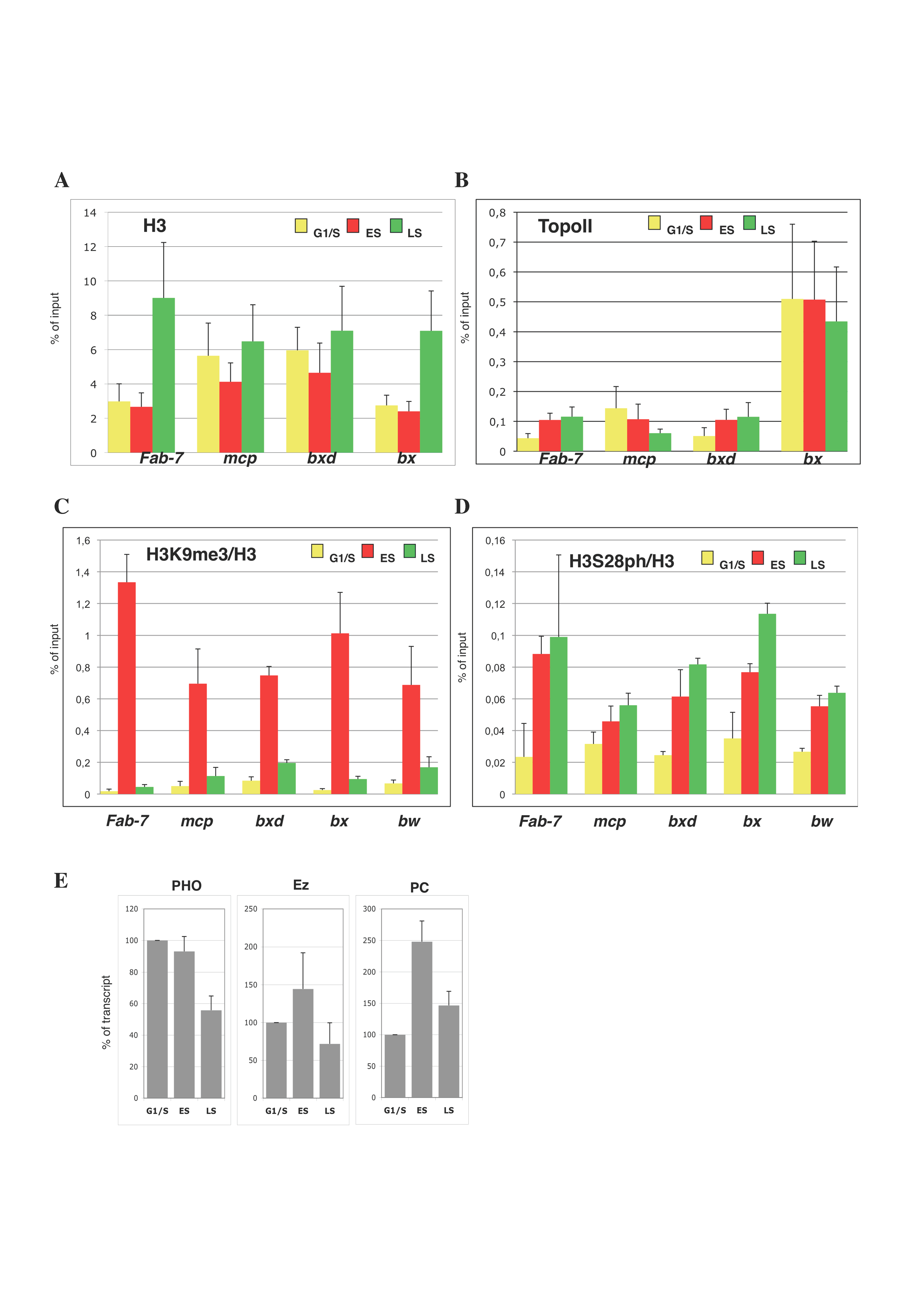

Supplement: Figure S2 — PcG dependent repressive mark are enriched at PREs before replication. (A–D) ChIP analysis with antibodies against H3 (A), Topoisomerase II (B), H3K9me3 (C) and H3S28ph (D) on synchronized cells. ChIP analysis are presented as percentage of input chromatin precipitated for each region. Mock enrichment is below 0.003% of the input. ChIP enrichment for H3 modifications are normalized to histone H3 density. Data obtained in HU treated cells (G1/S) are shown in yellow. Data obtained in cells collected 1 h and 2 h from HU block release (ES and LS) are in red and light green respectively. Each graph shows the result from at least three independent immunoprecipitation reactions done on different chromatin preparations. Standard error of the mean is indicated. (E) Quantification of transcription by qRTPCR. The transcription levels of PcG mRNA are shown as percentage of Gapdh expression. All data points were generated from the results of six independent experiments. Standard error of the mean is indicated. (TIFF) [file pgen.1002370.s002.tiff]

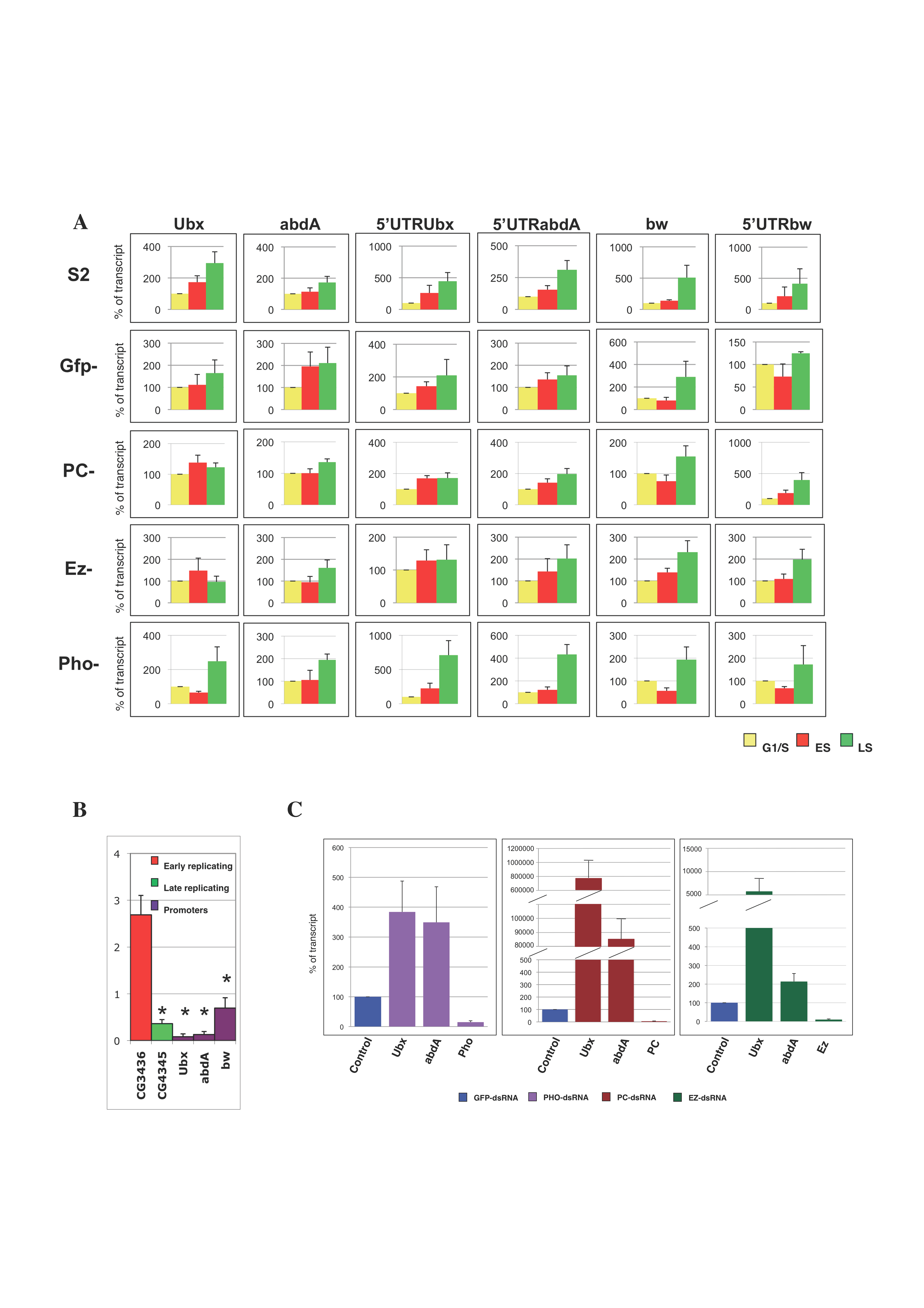

Supplement: Figure S3 — Transcriptional profile of homeotic genes during S phase progression is not affected by PcG proteins depletions. (A) Quantification of transcription levels of mature and primary transcripts of indicated genes by Real Time PCR in untreated S2 and Gfp-dsRNA, Pc-dsRNA, Pho-dsRNA or Ez-dsRNA treated S2 cells. Data obtained in HU blocked cells (G1/S) are shown in yellow. Data obtained in cells collected 1 h and 2 h from HU block release (ES and LS) are in red and light green respectively. Transcription levels are shown as percentage of Gapdh expression. No amplification was detected in the absence of RT. All data points were generated from the results of at least four independent experiments. Standard error of the mean is indicated. (B) Replication timing of analysed promoters as measured by qRTPCR. Ratios between the amplified products in early and late S phase are shown. We amplified positive controls for the early and late S phase and gene names correspond to their entries in FlyBase. All data points were generated from an average of at least three independent experiments. Standard error of the mean is indicated. Two-tailed t-test was applied for statistical analysis. Asterisks indicate statistically relevant differences; α = 0.05. P values: CG3436/CG4345: P = 0.0002; CG3436/Ubx promoter: P = 0.003; CG3436/abdA promoter: P = 0.004; CG3436/bw promoter: P = 0.01. (C) Quantification of transcripts by qRTPCR. Expression level of homeotic genes in GFP-RNAi S2 cells (blue), in PHO-dsRNA treated cells relative to GFP-RNAi S2 (violet), in PC-dsRNA treated cells relative to GFP-RNAi S2 (brown), in Ez-dsRNA treated cells relative to GFP-RNAi S2 (green). Transcriptional levels are shown as percentage of Gadph expression. All data points were generated from an average of four different experiments. Standard error of the mean is indicated. (TIFF) [file pgen.1002370.s003.tiff]

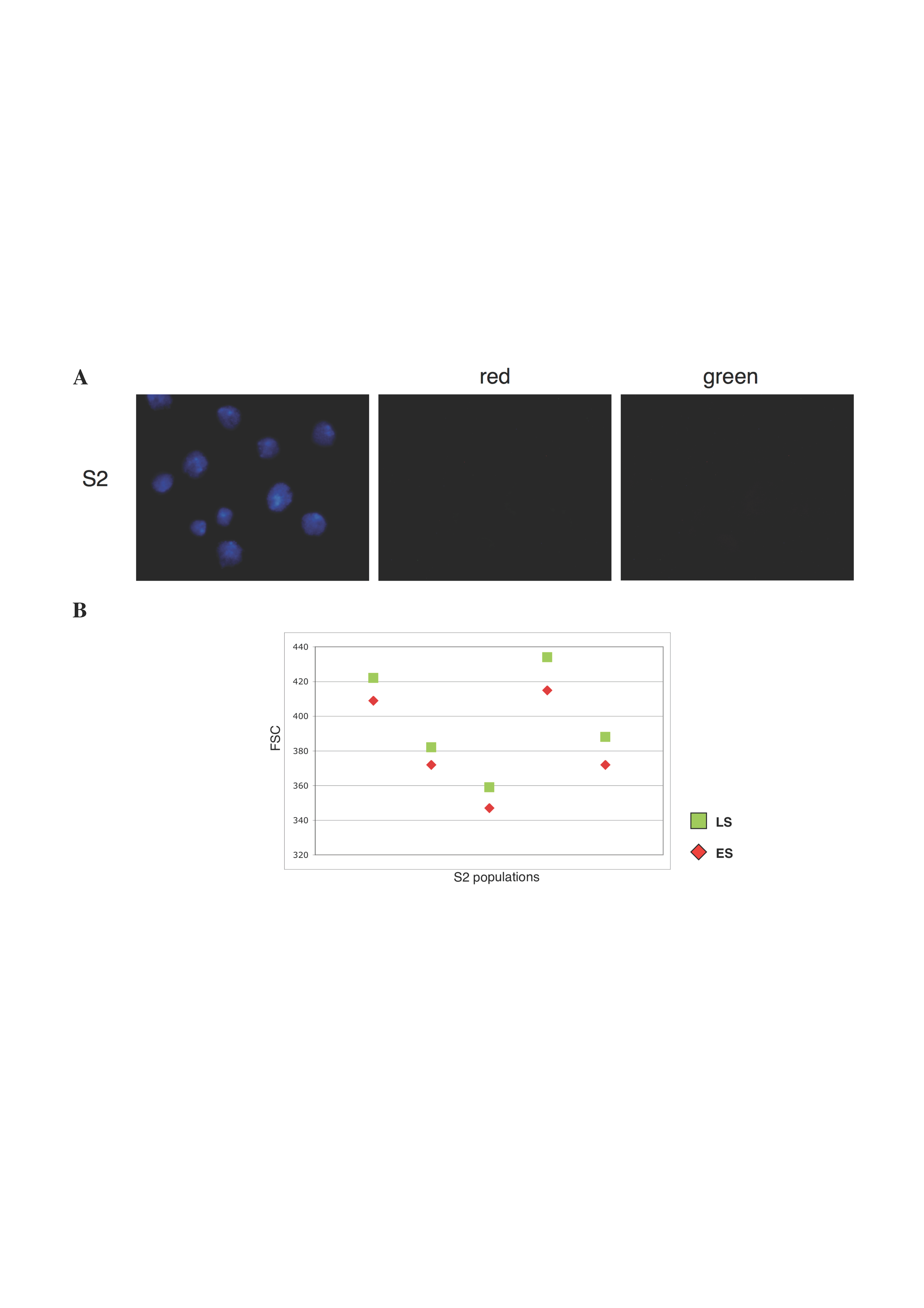

Supplement: Figure S4 — (A) Negative control of immunofluorescence experiment. Representative examples of S2 nuclei with double immunostaining using only secondary antibodies. (B) Cell dimensions in S phase measured by FACS. Dot plot indicating the mean FSC (Forward Scatter) of early (ES, red) and late S (LS, green) phase in 5 independent S2 populations. Student t test was applied for statistical analysis; α = 0.05. P = 0.0009. (TIFF) [file pgen.1002370.s004.tiff]
